# Clinically unfavorable transcriptome subtypes of non-WNT/non-SHH medulloblastomas are associated with a predominance in proliferating and progenitor-like cell subpopulations

**DOI:** 10.1007/s00401-024-02746-6

**Published:** 2024-06-07

**Authors:** Konstantin Okonechnikov, Daniel Schrimpf, Jan Koster, Philipp Sievers, Till Milde, Felix Sahm, David T. W. Jones, Andreas von Deimling, Stefan M. Pfister, Marcel Kool, Andrey Korshunov

**Affiliations:** 1 Hopp Children’s Cancer Center Heidelberg (KiTZ), Heidelberg, Germany; 2 Division of Pediatric Neuro-Oncology, German Cancer Research Center (DKFZ), German Cancer Consortium (DKTK), Heidelberg, Germany; 3 Clinical Cooperation Unit Neuropathology (B300), German Cancer Research Center (DKFZ), National Center for Tumor Diseases (NCT), German Cancer Consortium (DKTK), Im Neuenheimer Feld 280, 69120 Heidelberg, Germany; 4 Department of Neuropathology, Heidelberg University Hospital, Heidelberg, Germany; 5 Center for Experimental and Molecular Medicine, Amsterdam University Medical Centers, University of Amsterdam and Cancer Center Amsterdam, Amsterdam, The Netherlands; 6 Clinical Cooperation Unit Pediatric Oncology, German Cancer Research Center (DKFZ), German Consortium for Translational Cancer Research (DKTK), National Center for Tumor Diseases (NCT), Heidelberg, Germany; 7 Department of Pediatric Hematology and Oncology, Heidelberg University Hospital, Heidelberg, Germany; 8 Division of Pediatric Glioma Research, German Cancer Research Center (DKFZ), Heidelberg, Germany; 9 Princess Máxima Center for Pediatric Oncology, 3584CS Utrecht, The Netherlands; 10 University Medical Center Utrecht, Utrecht, the Netherlands; 11 National Center for Tumor Diseases (NCT), Heidelberg, Germany

**Keywords:** Medulloblastoma, Non-WNT/non-SHH, Subgroups, Deconvolution, Prognosis

## Abstract

The non-WNT/non-SHH (Grp3/Grp4) medulloblastomas (MBs) include eight second-generation subgroups (SGS; I–VIII) each with distinct molecular and clinical characteristics. Recently, we also identified two prognostically relevant transcriptome subtypes within each SGS MB, which are associated with unique gene expression signatures and signaling pathways. These prognostic subsets may be in connection to the intra-tumoral cell landscape that underlies SGS MB clinical-molecular diversity. Here, we performed a deconvolution analysis of the Grp3/Grp4 MB bulk RNA profiles using the previously identified single-cell RNA-seq reference dataset and focusing on variability in the cellular composition of SGS MB. RNA deconvolution analysis of the Grp3/Grp4 MB disclosed the subgroup-specific neoplastic cell subpopulations. Neuronally differentiated axodendritic GP3-C1 and glutamatergic GP4-C1 subpopulations were distributed within Grp3- and Grp4-associated SGS MB, respectively. Progenitor GP3-B2 subpopulation was prominent in aggressive SGS II MB, whereas photoreceptor/visual perception GP3/4-C2 cell content was typical for SGS III/IV MB. The current study also revealed significant variability in the proportions of cell subpopulations between clinically relevant SGS MB transcriptome subtypes, where unfavorable cohorts were enriched with cell cycle and progenitor-like cell subpopulations and, vice versa*,* favorable subtypes were composed of neuronally differentiated cell fractions predominantly. A higher than median proportion of proliferating and progenitor cell subpopulations conferred the shortest survival of the Grp3 and Grp 4 MB, and similar survival associations were identified for all SGS MB except SGS IV MB. In summary, the recently identified clinically relevant Grp3/Grp4 MB transcriptome subtypes are composed of different cell populations. Future studies should aim to validate the prognostic and therapeutic role of the identified Grp3/Grp4 MB inter-tumoral cellular heterogeneity. The application of the single-cell techniques on each SGS MB separately could help to clarify the clinical significance of subgroup-specific variability in tumor cell content and its relation with prognostic transcriptome signatures identified before.

**Supplementary Information:**

The online version contains supplementary material available at 10.1007/s00401-024-02746-6.

## Introduction

The non-WNT/non-SHH (Grp3/Grp4) medulloblastomas (MBs) comprise eight second-generation subgroups (SGS; I–VIII) each with distinct molecular and clinical characteristics [2, 7, 10]. This secondary refinement of the non-WNT/non-SHH MB cohort has improved its risk stratification, consequently making it more integrated as a standard procedure for patient inclusion in clinical trials [6]. Recently, we also identified two prognostically relevant transcriptome subtypes within each SGS MB which are associated with unique gene expression signatures and specific signaling pathways [5]. We also suggested that these prognostic subtypes may be in connection to the intra-tumoral cell landscape that underlies SGS MB clinical-molecular diversity; however, this hypothesis required further validation based on integration of information from advanced sequencing technologies.

The single-cell RNA sequencing is an appropriate data source to solve this task and it was already applied to decipher the cellular composition of various MB molecular groups wherein identified neoplastic cell subpopulations and transcriptome programs mapped to cerebellar cell lineages in the study by Hovestadt et al. [4]. In particular, the Grp3/Grp4 MBs included cell clusters that exhibited transcriptome signatures matching cell cycle/proliferation (program A), undifferentiated progenitors (program B), and neuronally differentiated cells (program C). Independently, Riemondy et al. [9] analyzed Grp3 and Grp4 MB samples at single-cell level and identified more fine-grained clusters of neoplastic cell populations: proliferating GP3-A, and GP4-A1/A2; progenitor GP3-B1/B2, and GP4-B1/B2; neuronally differentiated/glutamatergic GP3-C1/GP4-C1 and photoreceptor GP3-C2/GP4-C2 transcriptome cohorts.

The objective of the current study was to evaluate the intra- and inter-tumoral cell content within a large and uniformly processed non-WNT/non-SHH MB cohort using a deconvolution computational strategy, and thus focusing on differences in cellular composition of clinically relevant SGS MB transcriptome subtypes. For these purposes, we performed a deconvolution analysis of previously published Grp3/Grp4 MB bulk RNA transcriptome profiles [5] using the MB single-cell RNA-seq reference dataset [9] to impute the fractions of the cell populations and find potential associations with patients’ survival.

## Materials and methods

**Fig. 1:**
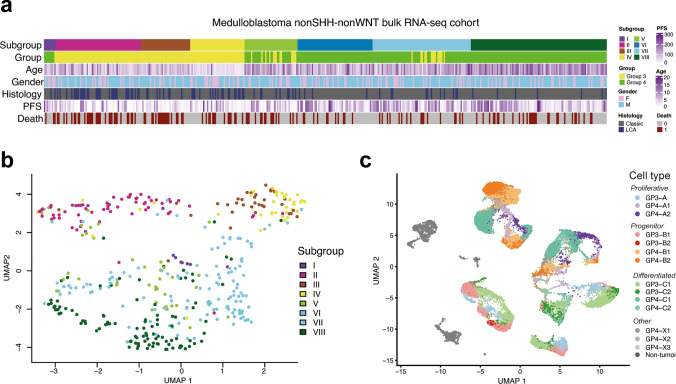
**a** Annotation heatmap of the target non-WNT/non-SHH MB bulk RNA-seq cohort (n = 435). **b** Uniform Manifold Approximation and Projection (UMAP) visualization of the target Grp3/Grp4 MB cohort based on the top 500 most highly variable genes which was subdivided by transcriptome profiling into eight second-generation MB subgroups. **c** UMAP visualization of cell composition generated by Riemondy et al. [9] with single-cell RNA-seq for 18 Grp3/Grp4 MB samples. Cell annotation is colored by defined clusters

Bulk RNA transcriptome profiles generated previously for clinically and molecularly annotated 435 non-WNT/non-SHH MB FFPE samples (Fig. 1a) were included focusing on previously preprocessed gene expression counts [5]. Uniform manifold approximation and projection (UMAP) visualization was performed on this dataset based on the usage of the top 500 most highly variable genes, demonstrating stability in inspection from 250 to 1500 (Fig. 1b). An additional set of snap-frozen bulk RNA samples (*n* = 168) was attracted from previously analyzed ICGC cohort [8], and 54 paired FFPE/ICGC Grp3/Grp4 MB samples were applied for comparison between deconvolution findings obtained for these cohorts.

Grp3/Grp4 MB cell signatures were extracted from previously published single-cell reference dataset [9] based on provided annotation, which was further used as the main source of cell types classification for Grp3/Grp4 MB. General visualization was performed via UMAP using the top 2500 most highly variable genes after normalization and the top ten principal components extraction based on the “elbow” cutoff method. The stability of the results was verified by inspection of highly variable genes selection from 1500 to 4000. Deconvolution analysis was performed with the BayesPrism tool [1] using the raw gene expression count matrices of the bulk dataset and of the corresponding MB single-cell RNA-seq dataset [9] as the reference to impute the fractions of the single-cell populations. Statistical evidence of a relative difference in cell type proportions between various SGS MB and prognostically relevant transcriptome subtypes was measured with a *t* test, afterward applying Benjamini–Hochberg correction per subgroup with a limit cutoff for an adjusted *p* value of 0.05. To verify the deconvolution results, gene set variance analysis (GSVA) [3] was performed on mean gene expression values computed from normalized matrices for target MB SGS sample cohorts with distinction on subgroups and favorable/unfavorable sample sets.

The distributions of PFS and OS were calculated according to the Kaplan–Meier method using the log-rank test and the result plots were created with R2: Genomics Analysis and Visualization Platform. For multivariate analysis, Cox proportional hazards regression models were used and estimated hazard ratios are provided with 95% confidence intervals. Statistical analyses were performed with R 3.5.1, with packages “survival”, “survminer” and “max stat” for uni- and multivariate survival analyses. A graphical overview of the study pipeline and applied methods are summarized in Suppl. Fig. 1a. Target bulk RNA-seq dataset as well as computed deconvolution results are publicly available at R2 platform (http://r2.amc.nl) under the title “Tumor medulloblastoma FFPE Group 3/4”.

## Results

### Neoplastic cell types composition within different MB SGS

In our study, we focused on bulk RNA-seq data from the Grp3/Grp4 MB cohort (*n* = 435) covering all non-WNT/non-SHH SGS MB to investigate intra-tumoral cellular heterogeneity and any association of this with clinical data. For this purpose, we used a published single-cell RNA-seq dataset [9] that was composed of Grp3 and Grp4 MB cases (*n* = 7 and *n* = 11, respectively) covering 14 clusters of neoplastic cells that exhibited molecular signatures matching different transcriptome programs (Fig. 1c). Deconvolution analysis of bulk RNA profiles was performed using the BayesPrism computational program (see Methods) and significant proportions of neoplastic cells (more than 90%) were identified in all tumor samples (Suppl. Fig. 1b–c). In particular, based on the analysis results from Riemondy et al. [9], the dataset was composed of three mitotic/proliferative cell types (GP3-A, GP4-A1/A2) associated with cell cycle pathways, four progenitor cell types (GP3-B1/B2, GP4-B1/B2) associated with ribosomal gene expression and four differentiated programs (GP3-C1/C2, GP4-C1/C2) with a variance in the associated pathways, e.g., GP3-C1 was enriched by genes related to axodendritic transport, GP4-C1 subpopulation distinguished by glutamate receptor pathway, while GP3-C2 and GP4-C2 subpopulations disclosed photoreceptor-associated signatures. A subset of neoplastic cell types (GP4-X1/X2/X3) was not classified in the reference study and had a low proportion (~ 3%); therefore, it was excluded from further analysis. The proportion of non-tumor glial and immune cells was quite low as well (~ 5%, Suppl. Fig. 1b–c), and therefore these cell types were also excluded.

**Fig. 2:**
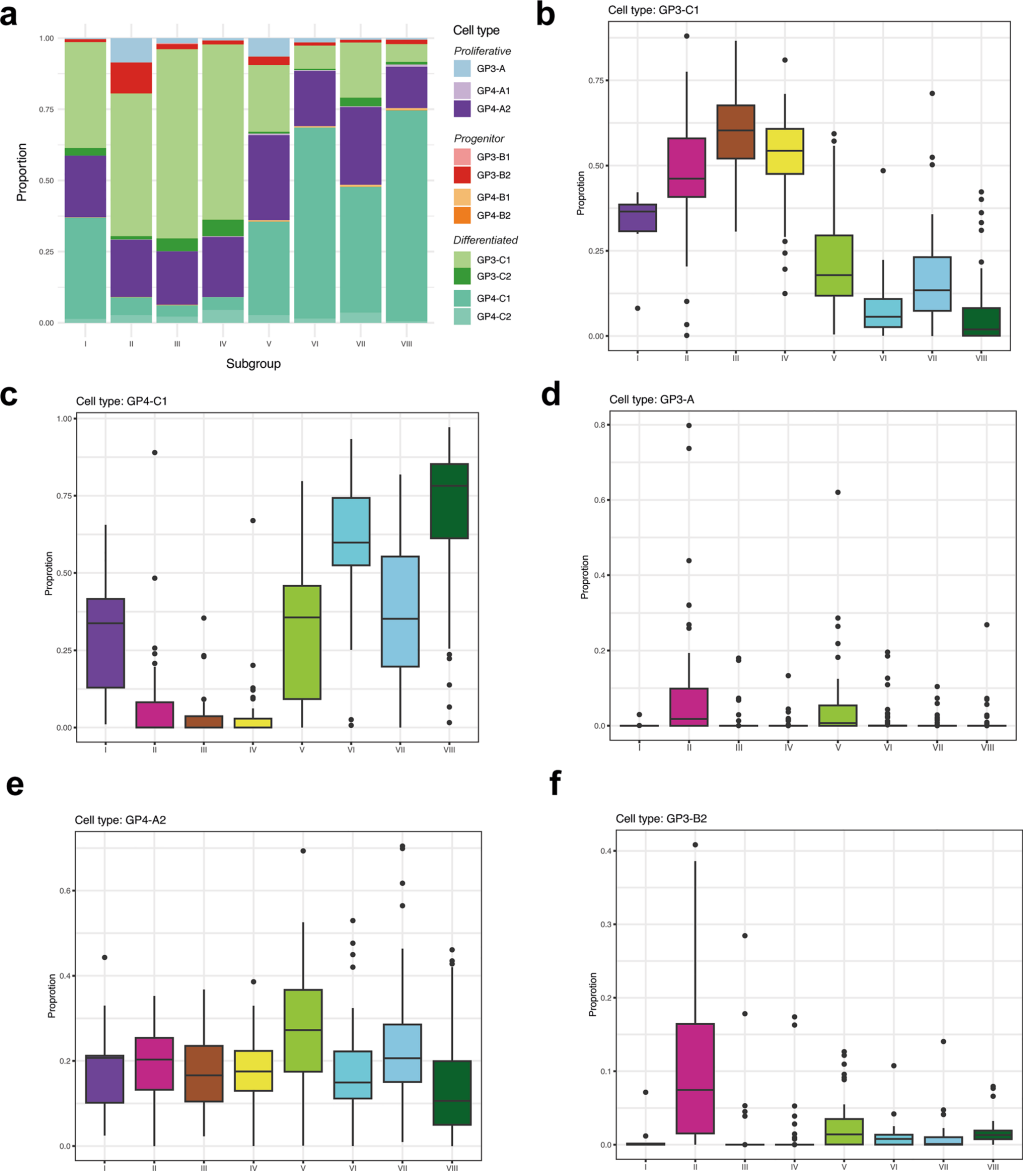
**a** Barplot of mean predicted Grp3/Grp4 MB cell type proportions across eight second-generation subgroups (SGS). Mean proportion values are computed per each subgroup. SGS MB II, III, and IV showed a high proportion of GP3-C1, but SGS MB VI and VIII revealed high GP4-C1 fraction. SGS MB I, V, and VII included mixed GP3-C1/GP4-C1 proportions. **b–f** Boxplot visualization of predicted cell type proportions for GP3-C1 **(b)**, GP4-C1 **(c)**, GP3-A **(d)**, GP4-A2 **(e)** and GP3-B2 **(f)**

In turn, various proportions of designated neoplastic cell subpopulations were identified within Grp3/Grp4 MB (Suppl. Fig. 1d-h) and each SGS MB analyzed (Fig. 2a). Thus, differentiated cell subpopulations demonstrated the largest proportions and revealed a significant association with the “consensus” Grp3 and Grp4 MB annotation [10]. In particular, Grp3-associated SGS MB II, III, and IV showed a high proportion of the GP3-C1 subpopulation (Fig. 2b), whereas Grp4-related SGS MB VI and VIII revealed larger proportions of the GP4-C1 cell fraction (Fig. 2c). However, SGS MB I, V, and VII, which are typically associated either with Grp3 or Grp4 MB signatures, correspondingly showed mixed proportions of GP3-C1/GP4-C1 neuronal subpopulations. Photoreceptor-associated GP3-C2/GP4-C2 signatures prevailed in SGS III/IV MB, thus reaching the highest cell proportion in SGS IV MB (~ 15%, Suppl. Fig.  2a–b). Proliferative GP3-A identified in SGS MB II and V (Fig. 2d), and GP4-A1 in SGS VIII (even though in low proportion, Suppl. Fig. 2c) were also subgroup-associated cell type populations. However, proportions of the GP4-A2 subpopulation did not differ between all eight SGS MB being independent of their allocation to the Grp3/Grp4 “consensus” MB groups (Fig. 2e). Progenitor-like cell GP3-B2 signature was associated strongly with SGS II MB and, to a lower degree, SGS V MB (Fig. 2f), whereas three other progenitor-associated cell subpopulations revealed only insignificant intra-tumoral proportions and did not differ between various SGS MB.

Identified group-associated distribution of various neoplastic cell subpopulations was also confirmed with the UMAP analysis (Suppl. Fig. 3). In line with a previous study [9], *MYC*-amplified MB (*n* = 31) included the highest proportions of proliferative GP3-A and progenitor GP3-B2 subpopulations as compared to the non-amplified MB cohort (data not shown). Also, higher proportions of proliferative and progenitor cell subpopulations were identified in advanced M2-3 stages for Grp3 MB, Grp4 MB, SGS V, and VII MB (data not shown).

To confirm our results independently, we also performed deconvolution analysis for our Grp3/Grp4 MB cohort by using GP3/GP4 transcriptome signatures identified in another single-cell RNA-seq study [4] that introduced cell cycle (program A), undifferentiated progenitor-like (program B), and neuronally differentiated (program C) cell types. Similar to the results from our main reference dataset, cell subpopulations connected to the cell cycle transcriptome program were evenly distributed among all these SGS MB, respectively (Suppl. Fig. 2d). In turn, progenitor-like signatures prevailed in Grp3-associated SGS II, III, and IV MB, (Suppl. Fig. 2e), whereas proportions of cells harboring neuronal program C prevailed across SGS I, V, VI, VII, and VIII MB (Suppl. Fig. 2f).

### Cell content differences in clinically relevant transcriptome SGS MB subtypes

**Fig. 3:**
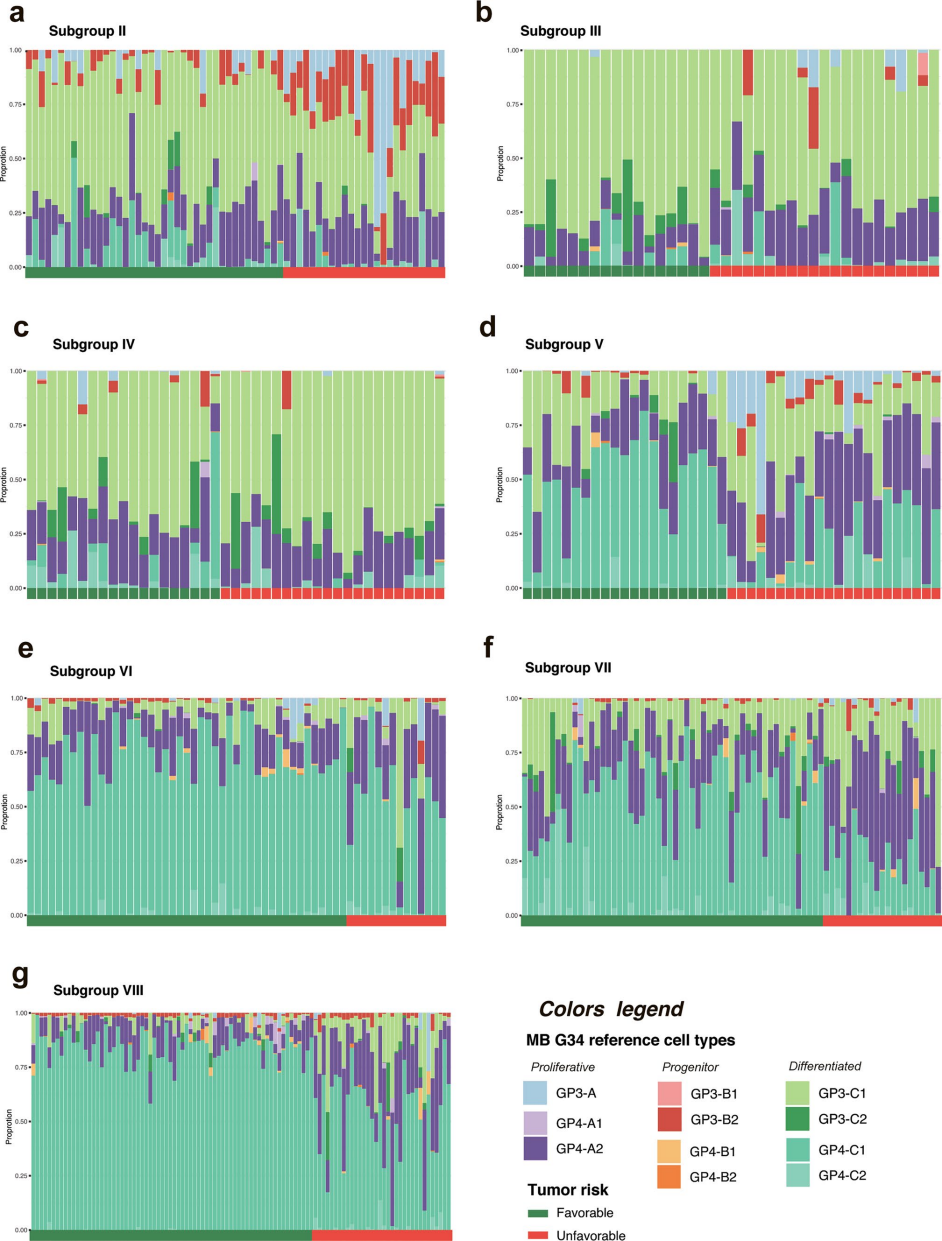
**a–g** Per sample barplot visualizations of predicted Grp3/Grp4 MB cell type proportions across second-generation subgroups subdivided into favorable (green bottom line) and unfavorable (red bottom line) transcriptome subtypes, respectively. Thus, unfavorable subtypes were enriched with cell cycle and progenitor-like cell subpopulations, and, vice versa*,* favorable transcriptome variants were composed of neuronally differentiated cell fractions

We further analyzed intra-tumoral cell content within recently identified clinically relevant subgroup-associated transcriptome subtypes [5]. In this analysis, the SGS II MB unfavorable subtype (Fig. 3a) showed higher proportions of proliferative GP3-A (Suppl. Fig. 4a) and progenitor-like GP3-B2 (Suppl. Fig. 4b) cell subpopulations, while, in contrast, the clinically favorable subset was composed of differentiated GP3-C1 subpopulation predominantly (Suppl. Fig. 4c). Similarly, in SGS III MB (Fig. 3b), proliferative subpopulation GP4-A2 was significantly higher in the unfavorable subset (Suppl. Fig. 4d). Notably, SGS IV MB did not show any statistically significant differences in neoplastic cell subpopulations between favorable and unfavorable transcriptome variants (Fig. 3c). SGS V MB unfavorable subtype (Fig. 3d) demonstrated increased proportions of proliferating GP3-A (Suppl. Fig. 4e) and GP4-A2 (Suppl. Fig. 4f) cell subpopulations combined with loss of differentiated GP4-C1 signature (Suppl. Fig. 4g). In SGS VI and VII MB (Fig. 3e–f), unfavorable subtypes showed increased proportions of proliferating subpopulation GP4-A2 (Suppl. Fig. 4h, j), whereas their favorable transcriptome variants were enriched with neuronal GP4-C1 signatures (Suppl. Fig. 4i, k). In contrast to other Grp4-associated SGS MB, the unfavorable SGS VIII MB transcriptome subtype (Fig. 3g) disclosed not only elevated proportions of proliferative GP4-A2 subpopulation (Suppl. Fig. 4l), but also tangible GP3-C1 cell fraction (Suppl. Fig. 4m). In contrast, the favorable SGS VIII transcriptome subtype was enriched with GP4-C1, but not GP3-C1 neuronal subpopulation (Suppl. Fig. 4n). Notably, high proportions of GP3-C1 and GP4-C1 subpopulations showed a strong negative correlation within SGS VIII MB (*r* = − 0.891; *p* < 0.01).

### Confirmation of obtained results by other methods

To verify the deconvolution results detected for the target non-WNT/non-SHH MB cohort, gene set variance analysis (GSVA) was performed as an alternative computational method on mean gene expression values computed from RPKM matrices generated for favorable and unfavorable transcriptome SGS MB subsets, as described (see Methods). GSVA results showed the enrichment patterns in expression signatures of the identified neoplastic cell subpopulations within the clinically relevant SGS MB subtypes, thus completely reflecting the results of bulk RNA deconvolution analysis (Suppl. Fig. 5a).

To exclude a possible sample-related bias, we also compared bulk tumor RNA deconvolution findings obtained for 54 Grp3/Grp4 MB paired FFPE/snap-frozen samples. The latter were previously included in the ICGC cohort thus applying by us as validation RNA-seq dataset generated from “fresh” tumor tissue [8]. We found a statistically significant concordance of tumor cell content between both these Grp3/Grp4 MB sample cohorts based on the positive correlation between obtained results for cells with mean proportion per sample > 10% e.g. GP3-A/GP3-C1(Suppl. Fig. 5b, c) and GP4-A2/GP4-C1 (Suppl. Fig5d, e).

## Survival analysis in SGS MB revealed associations with cell subpopulations

**Fig. 4:**
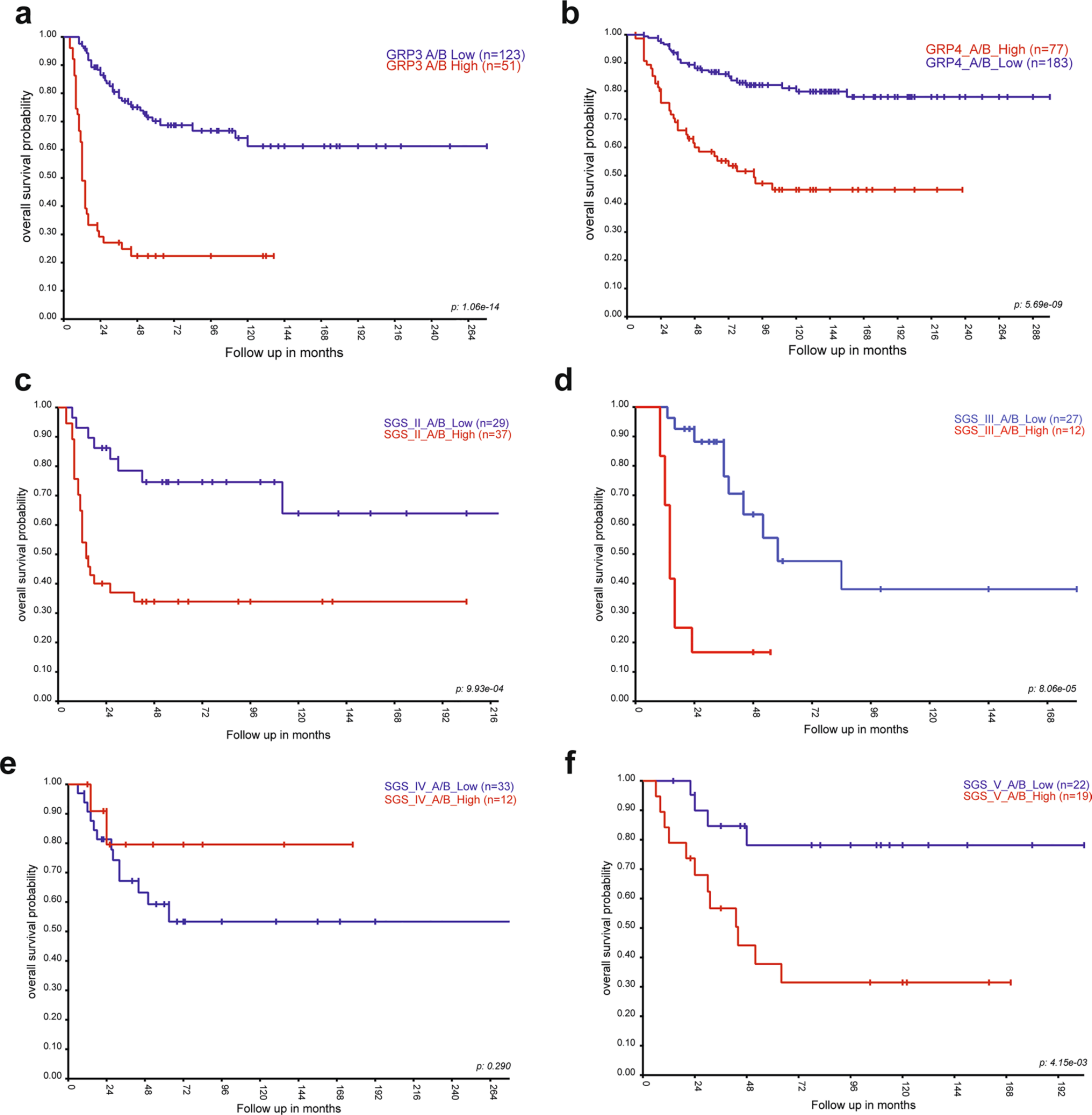
Kaplan–Meier overall survival curves with the impact of “low” and “high” A/B cell type proportions revealed prognostic significance for Grp3 MB **(a)**, Grp4 MB **(b)**, SGS II **(c)**, SGS III **(d)**, and SGS V **(f)**, but not SGS IV, **(e)** MB

**Fig. 5:**
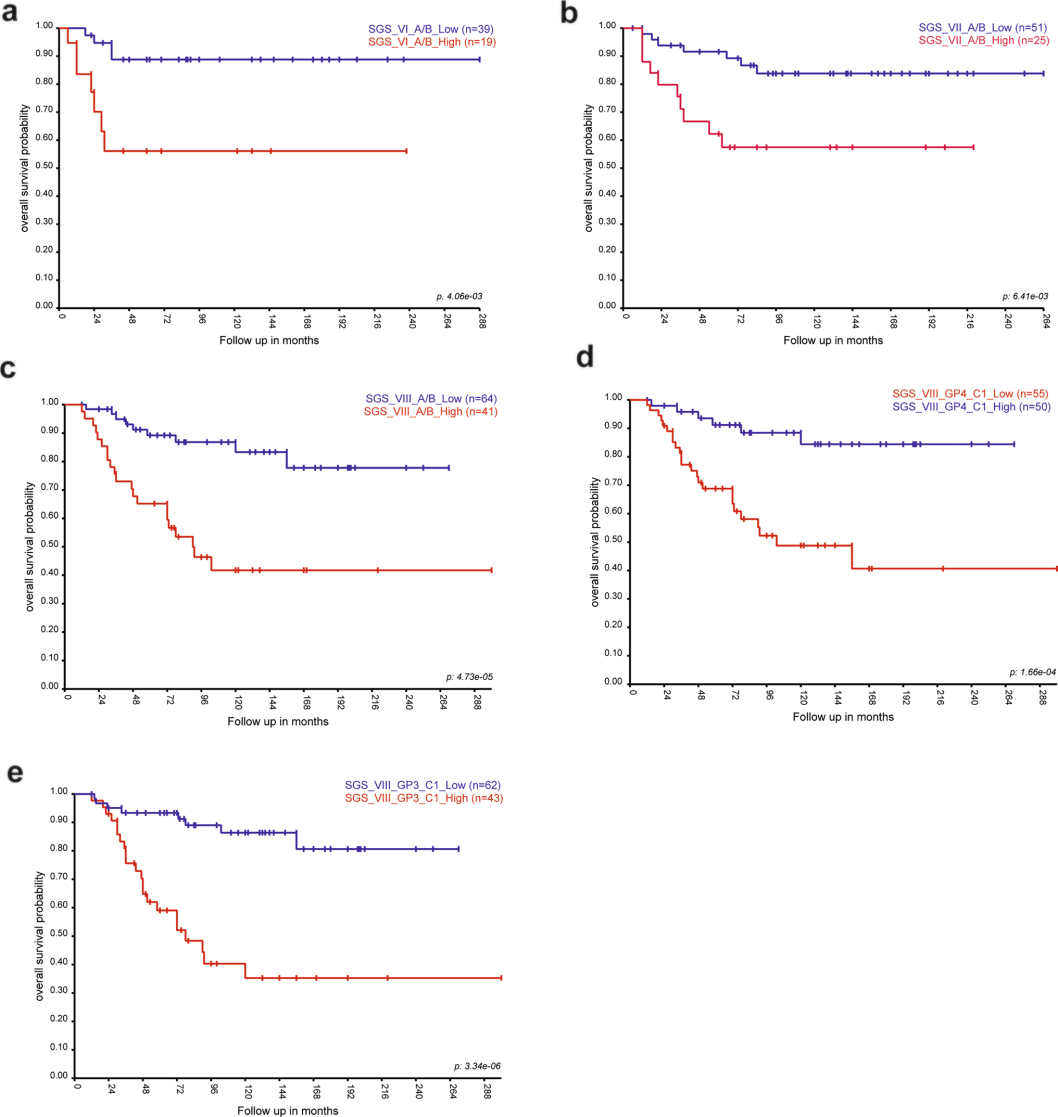
Kaplan–Meier overall survival curves with the impact of “low” and “high” A/B cell type proportions revealed prognostic significance for SGS VI **(a)**, SGS VII **(b)**, and SGS VIII **(c)**. “High” GP4-C1 **(d)** and GP3-C1 **(e)** cell type proportions revealed favorable and unfavorable prognostic significance for SGS VIII MB, respectively

A higher than median proportion of proliferating (combined GP3-A, GP4-A1/A2) and progenitor (combined GP3-B1/2 and GP4B1/B2) subpopulations conferred the shortest overall survival (OS; Fig. 4a, b) and progression-free survival (PFS; Suppl. Fig. 6a, b) of the Grp3 and Grp4 MB, respectively. Similar survival associations were identified for all SGS MB except SGS IV MB where higher proportions of unfavorable cell populations did not reach significant values for OS and PFS (Figs. 4c–f, 5a–c Suppl. Fig. 6c–i). Elevated proportions of neuronal GP3-C1/C2 subpopulations were associated with favorable outcomes for SGS II MB. Also, favorable survival significance for neuronal GP4-C1 signatures was identified for SGS VIII MB (Fig. 5d; Suppl Fig. 6j). In contrast, SGS VIII MB harboring higher than median proportions of the GP3-C1 subpopulation disclosed extremely poor survival (Fig. 5e; Suppl. Fig.  6k). Overall survival of other second-generation MB subgroups (III; IV, V, VI, and VII) was not associated significantly with median proportions of neuronally differentiated C1/C2 subpopulations. Multivariate analysis for different non-WNT/non-SHH MB groups (Table 1**)** and SGS **(**Table 2**)** was performed with various clinical and molecular variables including recently identified clinically relevant neoplastic cell subpopulations annotated to proliferating/progenitor transcriptome signatures. As a result, these prognostically unfavorable A/B cell signatures reached an independent level in the Cox regression model for Grp3 MB and SGS MB II, V, and VI, whereas clinically relevant transcriptome multigene signatures [5] were independent predictors for all included Groups and SGS MB.

**Table 1:** Multivariate analysis of PFS/OS for Group 3 MB (*n* = 175) and Group 4 MB (*n* = 260)

Variables	Group 3 MB PFS: HR/*p* value*	Group 3 MB OS: HR/*p* value *	Group 4 MB PFS: HR/*p* value *	Group 4 MB OS: HR/*p* value *
Age: infant vs. children	–	–	–	–
M stage: M2–3 vs. M0–1	–	–	2.21/ < 0.01	2.46/ < 0.01
Histology: large anaplastic vs. classic	–	–	–	–
Removal: GTR vs. NTR	–	–	–	–
Treatment: CHT vs. RT + CHT	–	–	–	–
*MYC* amplification: yes vs. no	–	1.84/ 0.03	–	–
*MYCN* amplification: yes vs. no	–	–	–	–
Whole chromosomal signature: yes vs. no	–	–	–	–
Isochromosome 17q: yes vs. no	–	–	–	–
Unfavorable transcriptome subtype**: yes vs. no	4.10 / < 0.01	6.26 / < 0.01	4.81 / < 0.01	6.44/ < 0.01
Unfavorable A/B cell signature: yes vs. no	2.23 / < 0.01	2.68 / < 0.01	–	–

*HR* hazard ratio; * *p* value log-rank test; **A.Korshunov et al., Acta Neuropathologica, 2023

**Table 2:** Multivariate analysis of PFS/OS for second-generation non-WNT/non-SHH MB subgroups (*n* = 435)

Variables	SGS MB II PFS/OS*	SGS MB III PFS/OS*	SGS MB IV PFS/OS*	SGS MB V PFS/OS*	SGS MB VI PFS/OS*	SGS MB VII PFS/OS*	SGS MB VIII PFS/OS*
Age: infant vs. children	–	–	–	–	–	–	–
M stage: M2–3 vs. M0–1	–	0.04 / 0.04	–	–	–	–	< 0.01/ < 0.01
Histology: large-anaplastic vs. classic	0.04/0.04	–	0.03 / 0.03	–	–	–	–
Removal: GTR vs. NTR	–	0.02 / -	–	–	–	–	–
Treatment: CHT vs. RT + CHT	0.04/ –	–	–	–	–	–	–
*MYC* amplification: yes vs. no	–	0.03 / 0.03	–	–	–	–	–
*MYCN* amplification: yes vs. no	–	–	–	0.03 / 0.03	–	–	–
Whole chromosomal signature: yes vs. no	–	–	–	0.01 / -	–	–	–
Isochromosome 17q: yes vs. no	–	–	–	–	–	–	–
Unfavorable transcriptome subtype**: yes vs. no	0.01 / 0.01	< 0.01 / < 0.01	< 0.01 / < 0.01	< 0.01 / < 0.01	< 0.01 / < 0.01	< 0.01/ < 0.01	< 0.01/ < 0.01
Unfavorable A/B cell signature: yes vs. no	0.03 / 0.03	–	–	0.02 / < 0.01	< 0.01 / < 0.01	–	–

*p value: log-rank test; **A.Korshunov et al., Acta Neuropathologica, 2023

## Discussion

Currently, numerous studies identified within non-WNT/non-SHH MB two “consensus” molecular groups designated as Grp3 and Grp4 MB with their further subdivision into multiple subgroups through I to VIII, which were included in the latest edition of the WHO CNS tumor classification [2, 5–7, 10–12]. On the other hand, it has been recently suggested that the non-WNT/non-SHH MB cohort represents a continuous spectrum of developmentally linked molecular subtypes [8, 11, 12]. In line with that concept, the current single-cell RNA studies revealed that the influence of MB cellular heterogeneity on their subgroup/subtype assignment precluded an elaboration of transcriptional markers to discriminate between designated molecular variants [9, 11, 12]. This indicates the imperfection of the existing non-WNT/non-SHH MB classification which requires further improvements and clarifications.

In the current study, bulk RNA deconvolution analysis of a non-WNT/non-SHH MB cohort applying a specific single-cell RNA-seq reference set [9] disclosed the existence of outlined neoplastic cell subpopulations within all SGS MB, although in various proportions. Thus, neuronally differentiated cell subpopulations were group-specific, because axodendritic GP3-C1 and glutamatergic GP4-C1 were distributed predominantly within Grp3- and Grp4-associated SGS MB, respectively. Progenitor GP3-B2 subpopulation accompanied by ribosomal signaling pathways [5] was prominent in clinically aggressive SGS II MB and closely associated with *MYC*-amplified tumors. In contrast, prognostically unfavorable SGS III MB disclosed a low content of progenitor-like cells, but harbored high proportions of proliferative subpopulations associated with DNA replication/cell cycle pathways [5]. Photoreceptor-differentiated C2 cell content was abundant in SGS III/IV MB, thus supporting the findings identified previously for these subgroups with gene ontology analysis [5]. However, the cell cycle GP4-A2 subpopulation was not group or subgroup specific, thus suggesting the existence of a non-specific proliferating cell cluster throughout the entire Grp3/Grp4 MB cohort. Potentially, this proliferating cell type could have additional molecular properties that were not covered in the currently available single-cell RNA experiments. More elaborated and detailed single-cell MB profiling could be an optimal resource in the future to decipher molecular signatures of this non-specific cell subpopulation and its clinical significance.

The current study also revealed significant variability in proportions of cell subpopulations between clinically relevant SGS MB transcriptome subtypes [5], where unfavorable cohorts were enriched with cell cycle and progenitor-like cell subpopulations and, vice versa*,* favorable subtypes were composed of neuronally differentiated cell fractions predominantly. These findings also illustrate the influence of MB intra-tumoral cellular composition on the development of prognostic SGS MB transcriptome subtypes, and, also, its potential clinical relevance based on bulk tumor sample deconvolution analysis.

Notably, the unfavorable SGS VIII transcriptome subset was enriched with neuronal GP3-C1 cell subpopulation which, in turn, was conversely correlated with glutamatergic GP4-C1 signature and associated with extremely poor survival. Thus, tumor cell subpopulation harboring Grp3-associated neuronal signatures could underlie biological aggressiveness for a tangible SGS VIII MB cohort and may be applied as a predictive molecular pattern for this MB variant that requires intense treatment upfront. Therefore, it would be important to elaborate on either single genomic marker or limited gene sets applying for the identification of the Grp3-associated SGS VIII MB subset defined initially with transcriptome and/or deconvolution techniques.

In summary, the current results indicate that the recently identified clinically relevant transcriptome subtypes within the non-WNT/non-SHH SGS MB are composed of different cell populations which can be uncovered with deconvolution analysis. Nevertheless, future studies should aim to validate the prognostic and therapeutic role of the identified Grp3/Grp4 MB inter-tumoral cellular heterogeneity. Thus, the application of the single-cell techniques on each SGS MB separately could help to clarify the clinical significance of subgroup-specific variability in tumor cell content and its relation with prognostic transcriptome signatures identified before.

## Supplementary Information

Below is the link to the electronic supplementary material.

Suppl. Fig. 1 a. Graphical overview of the study pipeline and applied methods. b. UMAP visualization of tumor, immune, and glial cell subpopulations generated with single-cell RNAseq for Grp3/Grp4 MB samples. Cell annotation is colored by defined clusters. c. Boxplot visualization of tumor (red), immune (gray), and glial (blue) cell proportions in Grp3/Grp4 MB cohort generated with bulk RNA deconvolution analysis. d-h) Boxplot visualization of GP3.A (d), GP4.A2 (e), GP3.B2 (f), GP3.C1 (g) GP4.C1 (h) cell type proportions with variance in Grp3 MB (yellow) and Grp4 MB (green). Suppl. Fig. 2. a–c Boxplot visualization of predicted cell type proportion from Riemondy et al [9] single cell dataset for GP3.C2 (a), GP4.C2 (b), and GP4.A1 (c). d–e Boxplot visualization of predicted cell type proportion from Hovestadt et al [4] single-cell dataset for Group 3.4.A (d), Group 3.4.B (e), and Group 3.4.C (f). Suppl. Fig. 3 UMAP visualization of various neoplastic cell subpopulations in Grp3/4 MB cohort. Most of the neoplastic cell subpopulations were annotated to corresponding “consensus” groups, but the proportions of the GP4-A2 subpopulation did not differ between them. Suppl. Fig. 4. a–o Boxplot visualization of predicted cell type proportion with variance in favorable (green) and unfavorable (red) SGS MB subtypes. The compared SGS MB cohorts and target cell types are annotated in the figure titles. Suppl. Fig. 5. a GSVA comparison figure for inspection of Grp3/4 MB cell type enrichment among favorable/unfavorable cases among Grp3/4 MB subgroups. b–e Comparison of deconvolution-derived cell type proportions among the same cases from FFPE RNA-seq (x386 axis) and fresh frozen RNA-seq (y-axis) for GP3.A (b) and GP3.C1 (c) across the Grp3 subset, for GP4.A2 (d) and GP4.C1 (e) across the Grp4 subset. Suppl. Fig. 6. Kaplan-Meier progression-free survival (PFS) curves with the impact of "low" and "high" A/B cell type proportions which revealed prognostic significance across Grp3 MB (a), Grp4 MB (b), SGS II (c), SGS III (d), SGS V (f), SGS VI (g), SGS VII (h), SGS VIII (i), but not for SGS IV (e). “High” GP4-C1 (k) and GP3-C1 (j) cell type proportions revealed favorable and unfavorable PFS for SGS VIII MB respectively. Supplementary file1 (PDF 2019 KB)

## Data Availability

Deconvolution results are publicly available at R2 platform (http://r2.amc.nl).
