# Supplementary figures and images for: Clinically unfavorable transcriptome subtypes of non-WNT/non-SHH medulloblastomas are associated with a predominance in proliferating and progenitor-like cell subpopulations
Source: Acta Neuropathol. 2024 Jun 7;147(1):95. doi: 10.1007/s00401-024-02746-6 (PMC12779650; doi:10.1007/s00401-024-02746-6)

**Suppl. Figure 1**

**a**

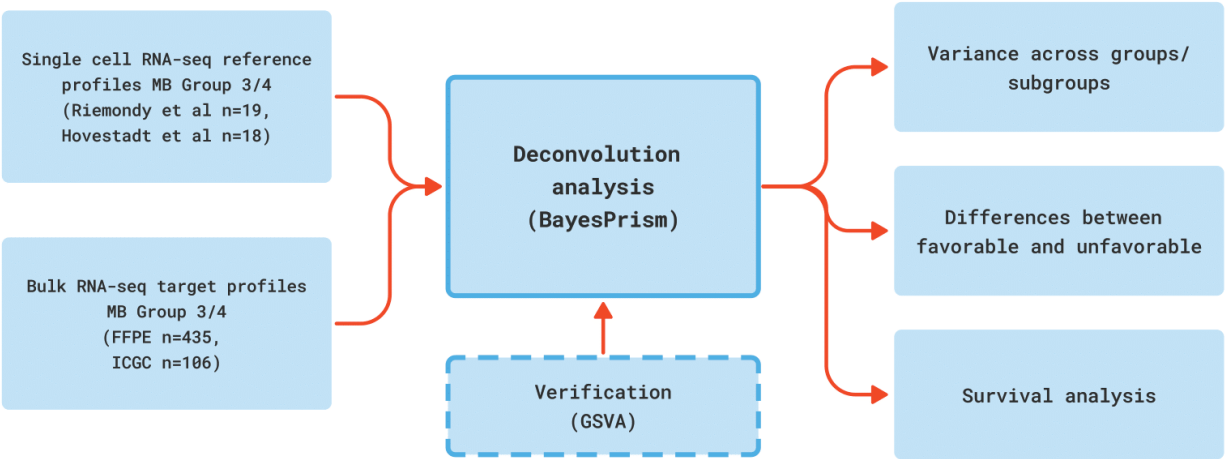

**b**

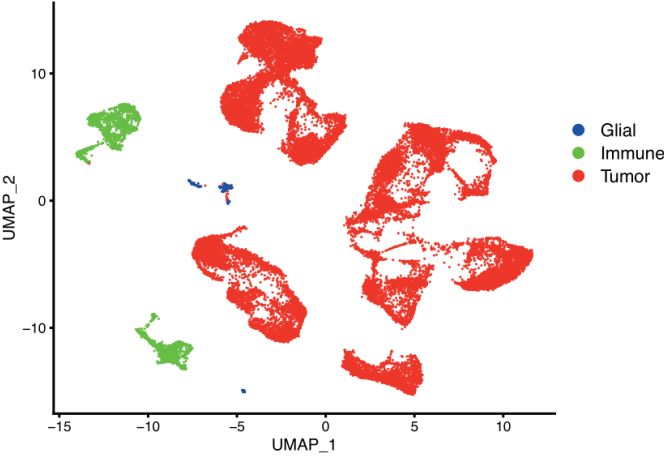

**c**

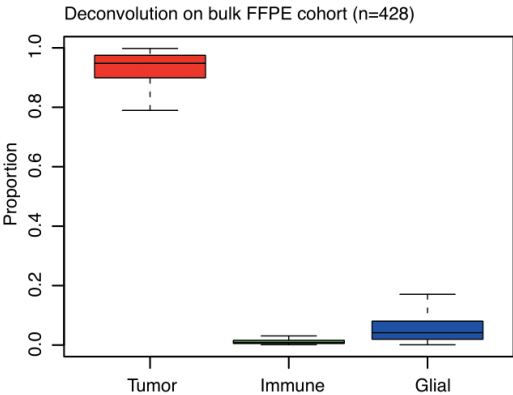

**d**

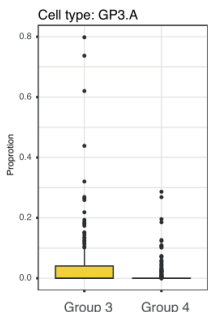

**e**

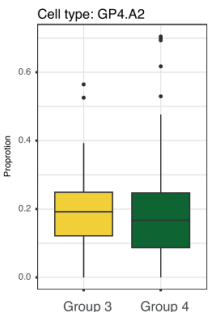

**f**

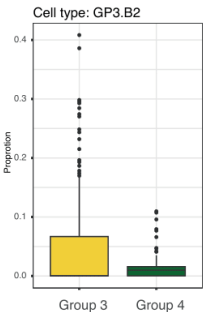

**g**

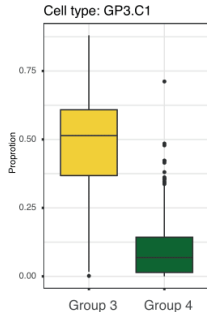

**h**

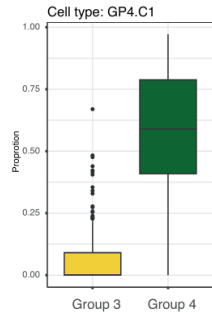

Suppl. Figure 2

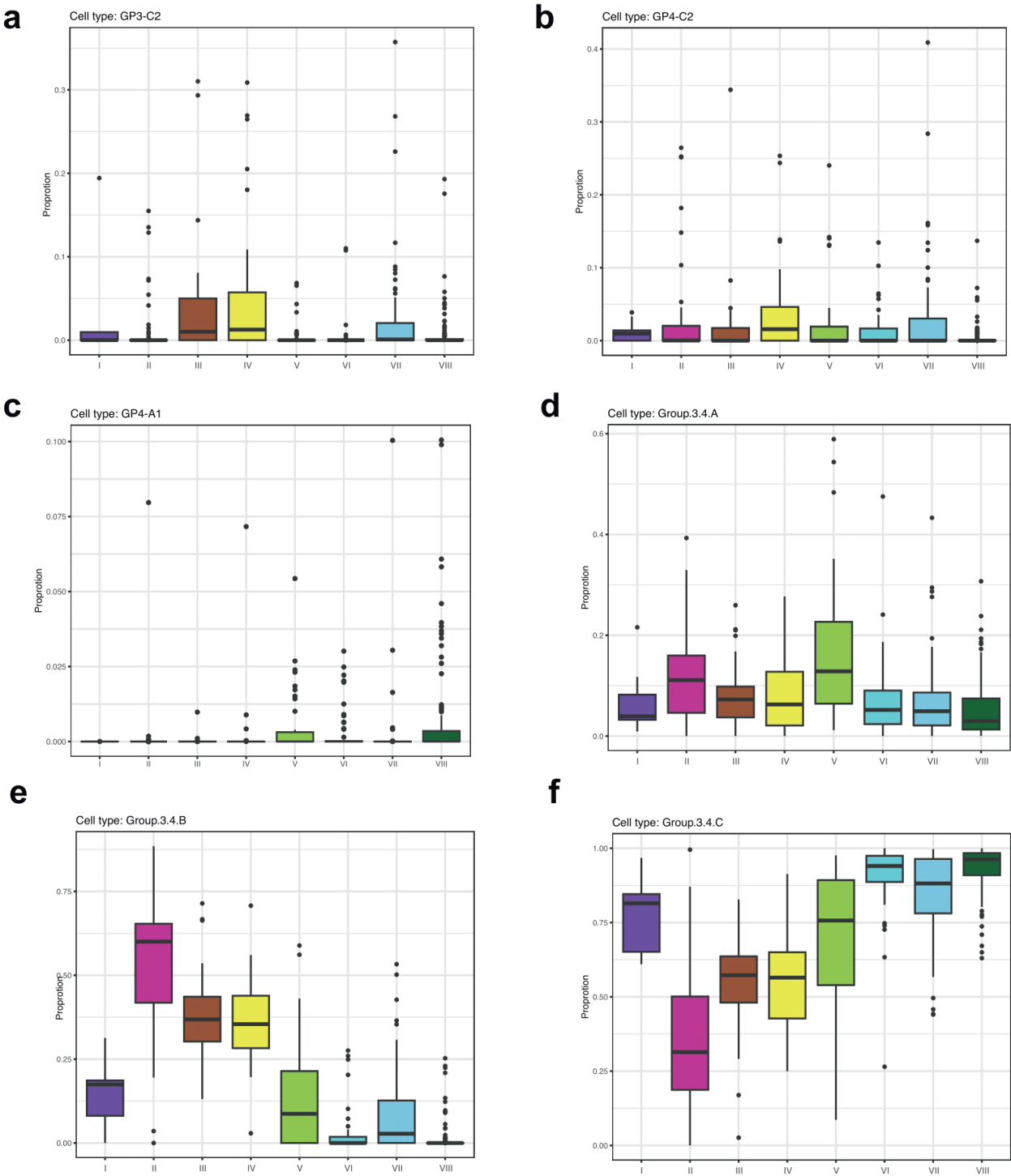

*Suppl. Figure 3*

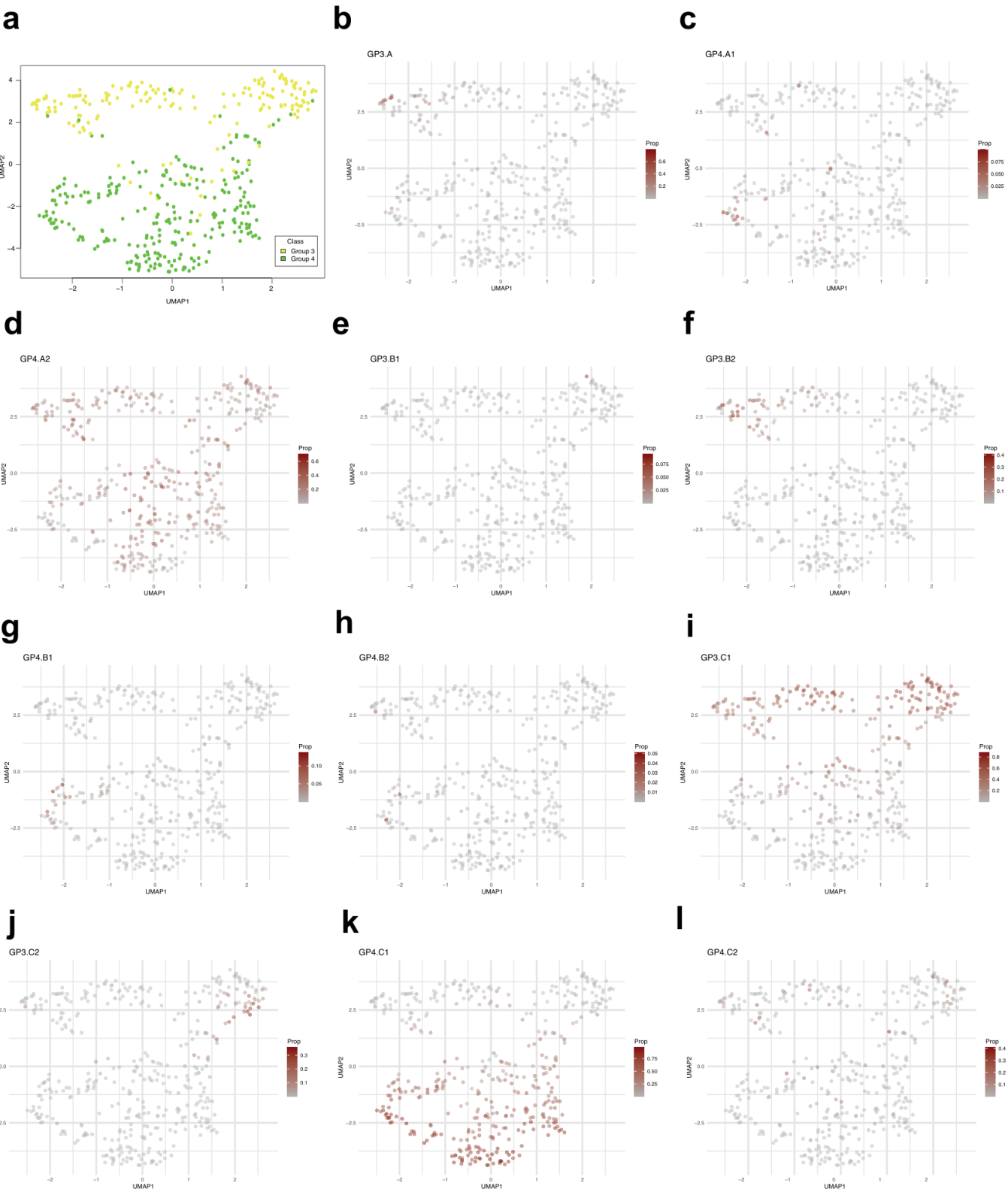

Suppl. Figure 4

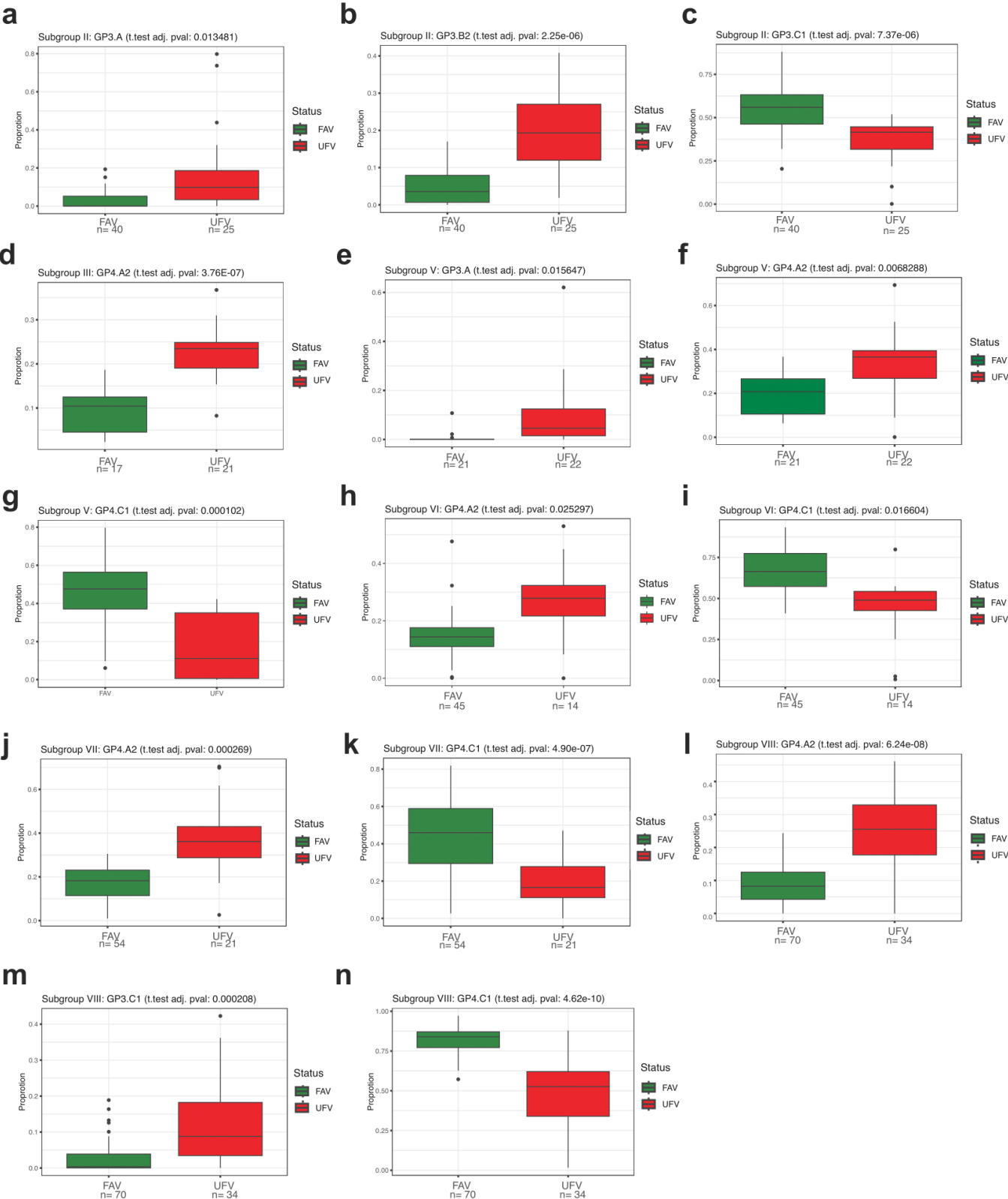

Suppl. Figure 5

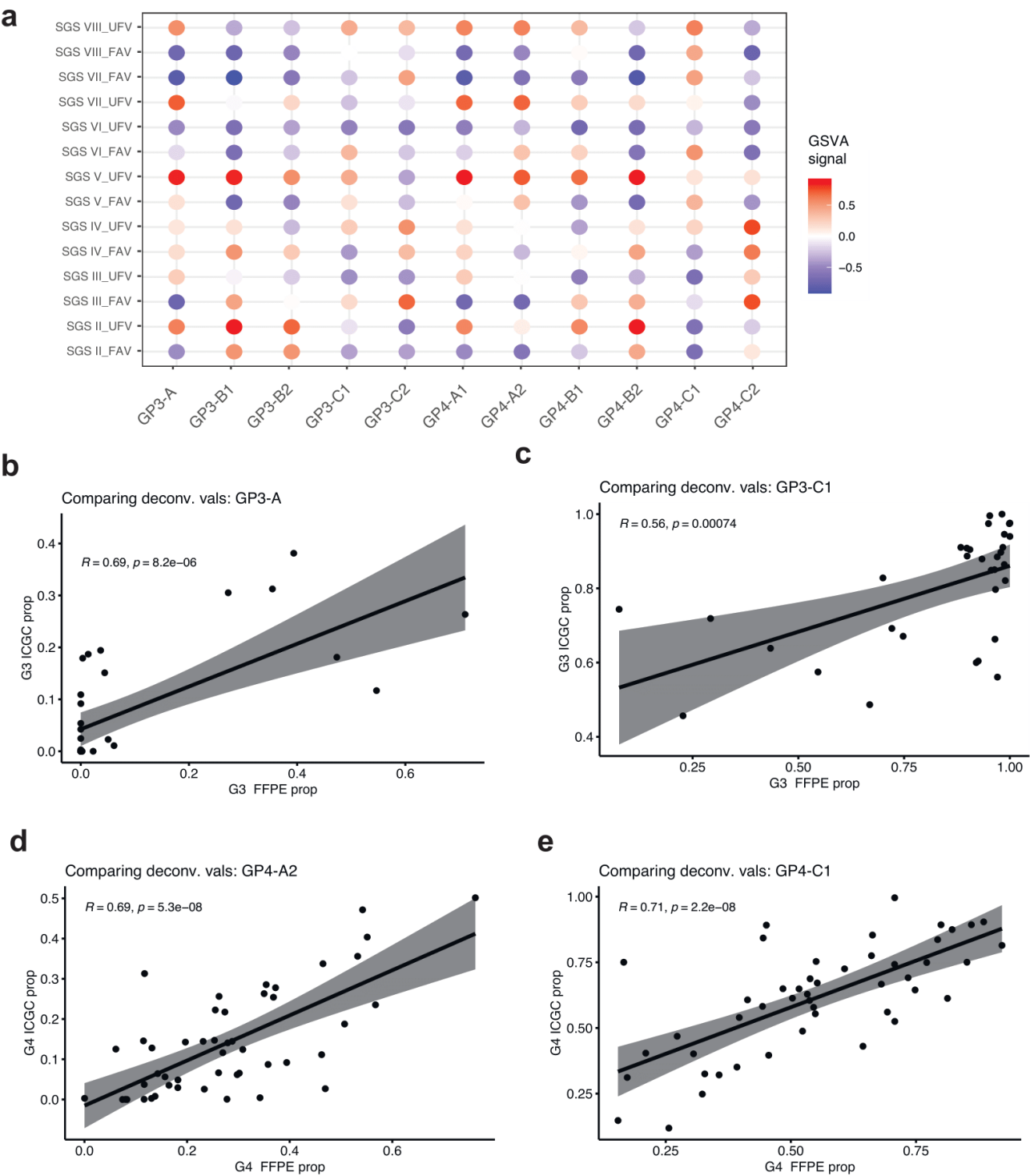

Suppl. Figure 6

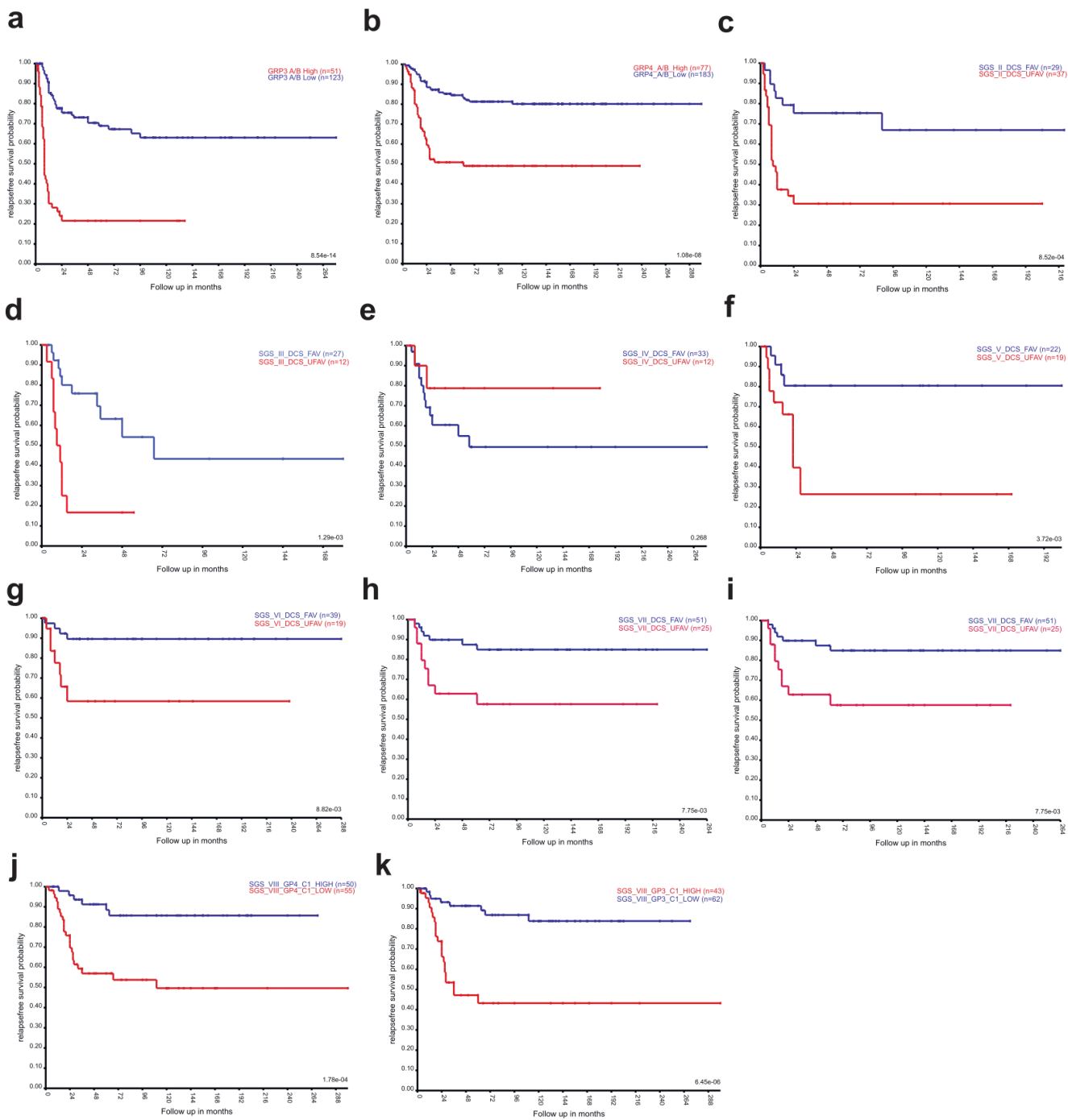

Supplement: Supplementary file 1 — Suppl. Fig. 1 a. Graphical overview of the study pipeline and applied methods. b. UMAP visualization of tumor, immune, and glial cell subpopulations generated with single-cell RNAseq for Grp3/Grp4 MB samples. Cell annotation is colored by defined clusters. c. Boxplot visualization of tumor (red), immune (gray), and glial (blue) cell proportions in Grp3/Grp4 MB cohort generated with bulk RNA deconvolution analysis. d-h) Boxplot visualization of GP3.A (d), GP4.A2 (e), GP3.B2 (f), GP3.C1 (g) GP4.C1 (h) cell type proportions with variance in Grp3 MB (yellow) and Grp4 MB (green). Suppl. Fig. 2. a–c Boxplot visualization of predicted cell type proportion from Riemondy et al [9] single cell dataset for GP3.C2 (a), GP4.C2 (b), and GP4.A1 (c). d–e Boxplot visualization of predicted cell type proportion from Hovestadt et al [4] single-cell dataset for Group 3.4.A (d), Group 3.4.B (e), and Group 3.4.C (f). Suppl. Fig. 3 UMAP visualization of various neoplastic cell subpopulations in Grp3/4 MB cohort. Most of the neoplastic cell subpopulations were annotated to corresponding “consensus” groups, but the proportions of the GP4-A2 subpopulation did not differ between them. Suppl. Fig. 4. a–o Boxplot visualization of predicted cell type proportion with variance in favorable (green) and unfavorable (red) SGS MB subtypes. The compared SGS MB cohorts and target cell types are annotated in the figure titles. Suppl. Fig. 5. a GSVA comparison figure for inspection of Grp3/4 MB cell type enrichment among favorable/unfavorable cases among Grp3/4 MB subgroups. b–e Comparison of deconvolution-derived cell type proportions among the same cases from FFPE RNA-seq (x386 axis) and fresh frozen RNA-seq (y-axis) for GP3.A (b) and GP3.C1 (c) across the Grp3 subset, for GP4.A2 (d) and GP4.C1 (e) across the Grp4 subset. Suppl. Fig. 6. Kaplan-Meier progression-free survival (PFS) curves with the impact of "low" and "high" A/B cell type proportions which revealed prognostic significance across Grp3 MB ( [file 401_2024_2746_MOESM1_ESM.pdf]
